# Determinants of COVID-19 Outcome as Predictors of Delayed Healthcare Services among Adults ≥50 Years during the Pandemic: 2006–2020 Health and Retirement Study

**DOI:** 10.3390/ijerph191912059

**Published:** 2022-09-23

**Authors:** Hind A. Beydoun, May A. Beydoun, Brook T. Alemu, Jordan Weiss, Sharmin Hossain, Rana S. Gautam, Alan B. Zonderman

**Affiliations:** 1 Department of Research Programs, Fort Belvoir Community Hospital, Fort Belvoir, VA 22060, USA; 2 Laboratory of Epidemiology and Population Sciences, National Institute on Aging Intramural Research Program, Baltimore, MD 21225, USA; 3 Health Sciences Program, School of Health Sciences, Western Carolina University, Cullowhee, NC 28723, USA; 4 Department of Demography, University of California Berkeley, Berkeley, CA 94720, USA; 5 Department of Sociology and Human Services, University of North Georgia, Dahlonega, GA 30597, USA

**Keywords:** cardiometabolic, coronavirus, socio-demographic, healthcare services, lifestyle, machine learning

## Abstract

Background: The coronavirus disease 19 (COVID-19) was declared a global pandemic on 11 March 2020. To date, a limited number of studies have examined the impact of this pandemic on healthcare-seeking behaviors of older populations. This longitudinal study examined personal characteristics linked to COVID-19 outcomes as predictors of self-reported delayed healthcare services attributed to this pandemic, among U.S. adults, ≥50 years of age. Methods: Secondary analyses were performed using cross-sectional data (1413 participants) and longitudinal data (2881 participants) from Health and Retirement Study (HRS) (2006–2018) linked to the 2020 HRS COVID-19 Project (57% female, mean age: 68 years). Demographic, socioeconomic, lifestyle and health characteristics were evaluated in relation to delayed overall, surgical and non-surgical healthcare services (“Since March 2020, was there any time when you needed medical or dental care, but delayed getting it, or did not get it at all?” and “What type of care did you delay”) using logistic regression and Ensemble machine learning for cross-sectional data as well as mixed-effects logistic modeling for longitudinal data. Results: Nearly 32.7% delayed healthcare services, 5.8% delayed surgical services and 31.4% delayed non-surgical services. Being female, having a college degree or higher and 1-unit increase in depression score were key predictors of delayed healthcare services. In fully adjusted logistic models, a history of 1 or 2 cardiovascular and/or metabolic conditions (vs. none) was associated with 60–70% greater odds of delays in non-surgical services, with distinct findings for histories of hypertension, cardiovascular disease, diabetes and stroke. Ensemble machine learning predicted surgical better than overall and non-surgical healthcare delays. Conclusion: Among older adults, sex, education and depressive symptoms are key predictors of delayed healthcare services attributed to the COVID-19 pandemic. Delays in surgical and non-surgical healthcare services may have distinct predictors, with non-surgical delays more frequently observed among individuals with a history of 1 or 2 cardiovascular and/or metabolic conditions.

## 1. Introduction

Caused by the Severe Acute Respiratory Syndrome Coronavirus 2 (SARS-CoV-2), the coronavirus disease 19 (COVID-19) was declared a global pandemic by the World Health Organization as of 11 March 2020 [1]. The time period preceding the advent of COVID-19 therapeutics and vaccines was characterized by surging COVID-19 cases, closures, an ill-equipped healthcare system, and limited lay and professional understanding of COVID-19. Subsequently, epidemiologic studies have identified advanced age, male sex, minority race/ethnicity, smoking, obesity and physical exercise as personal characteristics linked to SARS-CoV-2 positivity, COVID-19 morbidity and mortality risks [2,3,4,5,6]. Evidence from observational studies also suggests that cardiovascular and/or metabolic conditions may be predictive of worse prognosis for COVID-19 patients [3,4,5,6,7,8,9]. A systematic review and meta-analysis involving 4659 hospitalized COVID-19 patients identified from 14 published studies (1 January 2020–24 April 2020) suggested that mortality was strongly associated with hypertension (OR = 2.5, 95% CI: 2.1, 3.1), coronary heart disease (OR = 3.8, 95% CI: 2.1, 6.9) and diabetes (OR = 2.0; 95% CI: 1.7, 2.3) and that levels of C-reactive protein, interleukin-6, D-dimer, creatinine and alanine transaminase were higher and the level of albumin was lower among hospitalized COVID-19 patients who died versus those who survived [10]. The timely dissemination of COVID-19 information with regard to demographic, socioeconomic, lifestyle and health characteristics as predictors for SARS-CoV-2 positivity, COVID-19 morbidity and mortality risks as well as the link between cardiovascular and/or metabolic conditions and COVID-19 prognosis may have contributed to greater awareness and behavioral change including delayed healthcare services among COVID-19 high-risk populations [7,11]. Furthermore, public health strategies aimed at reducing person-to-person viral transmission including intermittent lockdowns constitute system-level barriers which may have contributed to delayed surgical and non-surgical healthcare services perceived to be unrelated to the global pandemic [6,7,12,13]. In particular, surgical services may have been delayed or canceled due to limited capacity or local mandates. 

Recent studies have evaluated the effect of COVID-19 pandemic healthcare delays on the diagnosis and treatment of a wide range of health problems, including cardiovascular disease [14,15,16,17,18,19,20,21] and cancer [22,23,24,25,26,27,28,29,30,31,32,33,34,35,36,37,38,39,40,41,42,43,44,45], with several studies focused on Emergency Department (ED), obstetrics, gynecology and pediatric patients [37,46,47,48,49,50,51,52,53] and a limited number of studies focused on surgical procedures [20,29,41,54,55,56,57,58,59,60,61,62,63,64]. To date, few studies have attempted to examine predictors of delayed surgical or non-surgical healthcare services in the general population [65,66]. Moreover, delayed healthcare services as a result of the COVID-19 pandemic among older populations—which are considered high-risk for COVID-19 as well as vulnerable to a wide range of health problems [16,67,68,69,70]—have rarely been evaluated. The objective of this longitudinal study is to examine the relationship of personal characteristics linked to COVID-19 outcomes as predictors of self-reported delayed overall, surgical and non-surgical healthcare services attributed to this pandemic among U.S. adults, ≥50 years of age, who participated in the Health and Retirement Study (HRS). Given the high level of evidence linking them to COVID-19 prognosis, we hypothesized that cardiovascular and/or metabolic conditions were independent predictors of outcomes of interest.

## 2. Materials and Methods

### 2.1. Data Source

The HRS is a longitudinal, population-based study that involves U.S. adults ≥50 years of age and spouses at any age which was designed to study labor force participation, economic well-being as well as health and family composition via biennial surveys administered through telephone or in-person interviews. Multistage probability sampling of U.S. households was conducted whereby specific groups were oversampled. All methods were carried out according to the declaration of Helsinki with written informed consent. The Institutional Review Board of the University of Michigan approved all study protocols, and HRS was sponsored by the National Institute on Aging and the Social Security Administration [67]. HRS details are reported elsewhere [68,69] and current analyses were determined research not involving human subjects at Fort Belvoir Community Hospital.

### 2.2. Study Participants

The original HRS study consists of cohorts whose data were collected during 1992, 1994 and 1996, and the Study of Asset and Health Dynamics of the Oldest Old (AHEAD) consists of those whose data were collected during 1993 and 1995. After these two studies were combined, two new cohorts, namely, the Children of the Depression and War Babies were added in 1998. Subsequently, Early Baby Boomers, Mid Baby Boomers and Late Baby Boomers were added in 2004, 2010 and 2016, respectively. Beginning in 2006, half of the HRS sample completed thorough in-person interviews whereas others completed a core telephone interview [70,71]. To reduce study-related costs and participant burden, enhanced interviewing alternated between distinct half-samples at each subsequent wave [70,71]. This sample was restricted to HRS participants for whom data were collected during the 2006–2018 waves and the 2020 COVID-19 wave, whereby an enhanced interviewing (EFTF) half-sample underwent telephone interviewing due to social distancing. Release to fieldwork occurred on 11 June 2020 (EFTF1) and 24 September 2020 (EFTF2). To meet our study objectives, we linked the latest release of the 2020 COVID-19 wave, which became available for 3266 EFTF participants in February 2021, with the 1992–2018 HRS longitudinal file provided by the RAND Center for the Study of Aging. 

**Figure 1:**
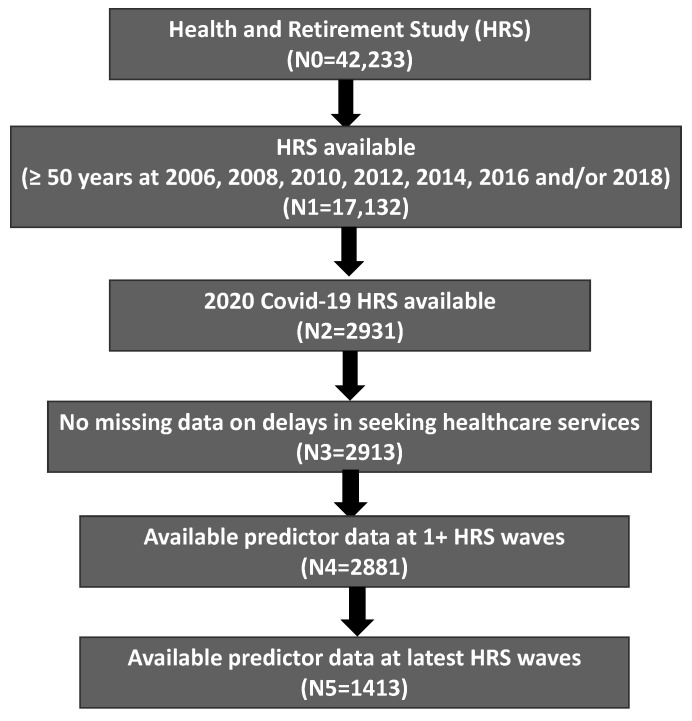
Study Flowchart.

A total of 17,132 of 42,233 HRS participants 50 years or older were identified at the 2006, 2008, 2010, 2012, 2014, 2016 and/or 2018 waves of data. Of those, 2931 participated in the 2020 COVID-19 project. Whereas 2913 HRS participants had data on delays in healthcare services, 2881 remained after excluding those with no predictor variables from at least one 2006–2020 HRS wave and 1413 remained after the exclusion of those with no predictor variables at the 2020 HRS wave (Figure 1). Accordingly, we analyzed longitudinal data on 2881 HRS participants (16,656 records) for mixed-effects logistic modeling and cross-sectional data on 1413 HRS participants from the 2020 COVID-19 project alone. Among 17,132 HRS participants who were ≥50 years of age at the 2006, 2008, 2010, 2012, 2014, 2016 and/or 2018 waves of data, 2881 with non-missing longitudinal data differed from 14,248 with missing data in terms of education (*p* < 0.0001), but not in terms of baseline age, sex, race or ethnicity. Similarly, 1413 with non-missing cross-sectional data differed from 15,719 with missing data in terms of sex (*p* = 0.026), race (*p* = 0.001) and education (*p* = 0.049), but not in terms of baseline age or ethnicity. 

### 2.3. Study Variables

The 2006–2020 HRS core data consist of standard questionnaire sections, which include the study items of interest. Moreover, the EFTF sub-samples had COVID-19 related items asked during the 2020 HRS wave. We assessed several demographic, socioeconomic, lifestyle and health characteristics, at multiple 2006–2020 HRS waves, taking into account available repeated measurements for all predictor variables besides sex, race, ethnicity and education. Specifically, we defined 7 characteristics identified in the published literature as predictors of COVID-19 outcomes and that may consequently predict delays in healthcare services attributed to the COVID-19 pandemic. We identified 12 additional characteristics that may confound or modify hypothesized associations between these characteristics and delays in healthcare services.

#### 2.3.1. COVID-19 Determinants

##### Demographic, Socioeconomic and Lifestyle Characteristics

HRS data were extracted on sex, birth cohort, age, race, ethnicity, marital status, education, work status, total wealth (U.S. dollars), number of household members and Census region of residence [72]. HRS data were also extracted on smoking status, frequency of alcohol consumption and frequency of moderate and vigorous exercise. Whereas most socio-demographic and lifestyle variables were available until 2020, marital status, number of household members and region of residence were available with little missing data by 2018, whereby missing 2020 data was imputed with information from a previous wave. 

##### Health Characteristics

Self-report weight and height as well as cardiovascular and/or metabolic conditions were obtained from 2006–2020 HRS data. Body mass index (BMI) was calculated as weight (in kilograms) divided by height (in meters) squared, and categorized as <25, 25–29.9, ≥30 kg/m^2^. The presence of cardiovascular and/or metabolic conditions was determined through physician-diagnosed hypertension, diabetes, heart disease and/or stroke. The total number of cardiovascular and/or metabolic conditions was further categorized as ‘0’, ‘1–2’ and ‘≥3’ [69,73]. Self-rated health was evaluated based on a single item and further dichotomized as ‘excellent/very good/good’ or ‘fair/poor’. Depressive symptoms were evaluated using a modified 8-item Center for Epidemiological Studies Depression Scale (CES-D) and total CES-D score was calculated whereby higher scores suggested worse depressive symptoms [69,73]. 

#### 2.3.2. Delays in Healthcare Services

Self-reported delays in healthcare services due to the COVID-19 pandemic was determined using a series of dichotomous (‘yes’ or ‘no’) questionnaire items as part of the 2020 HRS COVID-19 wave initiated on 11 June 2020 for EFTF1 participants and on 24 September 2020 for EFTF2 participants. These questionnaire items were worded as follows: “Since March 2020, was there any time when you needed medical or dental care, but delayed getting it, or did not get it at all?” and “What type of care did you delay...” with several probing questions focused on delayed ‘surgery’, ‘seeing the doctor’ [including telemedicine], ‘filling a prescription’, ‘dental care’ and/or ‘other type of care’. Delays in surgery were further distinguished from other types of healthcare services.

### 2.4. Statistical Analysis

Stata release 16 (StataCorp, College Station, TX, USA) and R release 4.0.2 (R Foundation for Statistical Computing, Vienna, Austria) were used to perform complete subject analyses taking into consideration the complex sampling design with a preliminary weight variable called CVWGTR [70,71]. Categorical data were summarized using counts and percentages and continuous data were summarized using mean, median, standard error of the mean (SEM) and interquartile range, as appropriate. We performed uncorrected Chi-square and design-based F-tests to evaluate bivariate associations as well as traditional regression and machine learning (ML) for predictive modeling. Initially, we described cardiovascular and/or metabolic conditions, demographic, socioeconomic, lifestyle and health characteristics at the last available wave of data collection according to delayed healthcare services. Afterwards, we examined BMI as well as cardiovascular and/or metabolic conditions as predictors of delayed healthcare, prior to and after controlling for demographic, socioeconomic, lifestyle and health characteristics. Specifically, logistic models were generated for cross-sectional data, and mixed-effects logistic models were generated for longitudinal data. In these analyses, Models I were adjusted for demographic and socioeconomic characteristics (sex, birth cohort, age, race, ethnicity, marital status, education, work status, federal insurance coverage, total wealth, number of household members, Census region of residence), Models II were adjusted for demographic, socioeconomic and lifestyle (smoking status, frequency of alcohol consumption, frequency of moderate and vigorous exercise) characteristics and Models III were adjusted for demographic, socioeconomic, lifestyle and health (self-rated health, depressive symptoms) characteristics. Third, predictive models for delayed overall, surgical and non-surgical healthcare were constructed. After excluding predictor variables that were not related to outcomes of interest at α = 0.2, the remaining predictor variables were entered into logistic and mixed-effects logistic models. Finally, as an alternative methodology to logistic regression, ML algorithms were applied to identify the best predictive model for delays healthcare services using cross-sectional data and based on specific performance criteria [74,75,76]. ML algorithms are more flexible than regression techniques as they can manage several covariates while evaluating non-linear relationships and interaction effects, with the potential for superior predictive performance. SuperLearner is an Ensemble ML algorithm for estimating performance of candidate models called “learners” and creating an optimally weighted average for algorithms or “Ensemble” using specific performance criteria (e.g., cross-validated area under the receiving operating characteristic curve (cv-AUROC), cross-validated Risk (cv-Risk) or error in predicting outcomes) [74,75,76,77]. In this study, we applied an algorithm that relies on multiple ML algorithms (Least Absolute Shrinkage and Selection Operator (LASSO), Random Forests, XGBoost and Support Vector Machines (SVM)), a V-fold “inner” cross-validation, a U-fold “outer” cross-validation and a loss function for the identification of the best weighted combination of prediction models [74,75,76,77,78,79]. As described in previous studies [74,75,76,77,80], ~80% of HRS participants were incorporated into a training sample and ~20% of HRS participants were incorporated into a test sample. Two-sided statistical tests were performed at a significance level α = 0.05.

## 3. Results

Table 1 displays distributions of demographic, socioeconomic, lifestyle and health characteristics in relation to delayed healthcare services (overall, surgical and non-surgical) at the 2020 HRS wave. Approximately 57.2% of study participants were female, with a mean age of 67.5 years, 76.5% were White or Caucasian, 23.3% had a college degree or higher, 66.5% were not working, 69.0% reported federal health insurance coverage and 32.4% reported a total wealth <USD 25,000. Nearly 41.1% of study participants resided in the Southern region of the United States and the mean number of household members was 2.2. Furthermore, 40.8% were never smokers, 42.6% abstained from alcohol consumption and 54.6% performed moderate or vigorous physical activity more than 1 time per week. Approximately 43.1 were obese and 14.1% had ≥3 co-morbid conditions. Additionally, 32.7% delayed healthcare services, 5.8% delayed surgical services and 31.4% delayed non-surgical services. Females were more likely than males to delay healthcare services (odds ratio (OR) = 1.48, 95% confidence interval (CI): 1.07, 2.06), in general, and non-surgical healthcare services (OR = 1.47, 95% CI: 1.05, 2.05), in particular. By contrast, delays in surgical and/or non-surgical healthcare were more prevalent among ‘Mid Baby Boomers’ and ‘Late Baby Boomers’ when compared to the ‘Original’, ‘AHEAD’ or ‘Children of the Depression’ birth cohorts. Age was inversely related to delays in surgical and/or non-surgical and individuals between the ages of 55 and 64 years were more likely than those < 55 years of age to delay surgical healthcare. There was a notable dose–response relationship between delaying healthcare and increasing level of education, whereby those with a college degree or higher were more likely to delay healthcare, in general, and non-surgical healthcare, in particular, as compared to those with no degree. A similar pattern was observed whereby working individuals were nearly 50% more likely than non-working individuals to delay non-surgical healthcare. Individuals who consumed alcohol 1–3 days per month were more likely than abstainers to delay surgical and/or non-surgical healthcare, and current smokers were nearly twice as likely to delay surgery as compared to never-smokers. Fair or poor health also predicted overall delay of healthcare as opposed to excellent/very good/good self-rated health (OR = 1.43, 95% CI: 1.03, 1.97). Most notably, depressive symptom score (per unit increase) was a strong predictor for delaying healthcare in all three categories (Overall: OR = 1.15, 95% CI: 1.07, 1.24; Surgical: OR = 1.17, 95% CI: 1.04, 1.33; Non-surgical: OR = 1.14, 95% CI: 1.06, 1.24).

Table 2 presents multiple logistic and mixed-effects logistic models for cardiovascular and/or metabolic conditions as predictors of delays in healthcare services before and after controlling for demographic, socioeconomic, lifestyle and health characteristics. In the logistic model adjusted only for demographic and socioeconomic characteristics, having 1–2 cardio-metabolic risk factors and/or chronic conditions was associated with increased risk of delays in healthcare (OR = 1.73, 95% CI: 1.13, 2.65), an association that was only slightly changed by addition of lifestyle characteristics (OR = 1.74, 95% CI: 1.15, 2.65) or in the fully adjusted model (OR = 1.62, 95% CI: 1.06, 2.49). When examining delayed surgical (Table 3) and non-surgical (Table 4) healthcare services, having 1–2 cardio-metabolic risk factors and/or chronic conditions was associated with an increased odds of delayed non-surgical healthcare in Model I (OR = 1.75, 95% CI: 1.14, 2.69), Model II (OR = 1.79, 95% CI: 1.16, 2.72) and Model III (OR = 1.68, 95% CI: 1.08, 2.59), but not delayed surgical healthcare. These associations were not statistically significant when using mixed-effects logistic models. By contrast, fully adjusted mixed-effects logistic models suggested that individuals with a history of cardiovascular disease were less likely to delay surgeries, whereas those with a history of diabetes or stroke were more likely to delay surgeries. Finally, delays in non-surgical healthcare were less frequent among individuals with a history of hypertension (Table 2, Table 3 and Table 4).

After removing demographic, socioeconomic, lifestyle and health characteristics not significantly related to delayed healthcare services at α = 0.2 in Table 1, the remaining variables were entered into logistic and mixed-effects logistic models for predicting delays in surgical and/or non-surgical healthcare services (Table 5). Being female, having a college degree or higher and a 1-unit increase in depressive symptom score were consistent independent predictors of overall and non-surgical delays in healthcare services in logistic and mixed-effects logistic models. Although entered into these regression models, histories of diabetes and stroke were not consistently associated with outcomes of interest, after controlling for other covariates.

Table 6 presents the outcome of the SuperLearner model for cross-sectional predictors of COVID-19 healthcare delays, using four distinct ML algorithms. Whereas ‘LASSO’ had the lowest cv-Risk suggesting less error in predicting the outcomes of interest, ‘XGBoost’ had the highest cv-Risk for the overall and non-surgical outcomes and ‘SVM’ had the highest cv-Risk for the surgical outcome. The weighted average cv-Risk for the SuperLearner model was 0.214 for the overall outcome with cv-AUROC = 0.600. The weighted average cv-Risk for the SuperLearner model was 0.211 for the non-surgical outcome with cv-AUROC = 0.655. Conversely, the weighted average cv-Risk for the SuperLearner model was 0.052 for the surgical outcome with cv-AUROC = 0.920. Accordingly, the SuperLearner model was highly predictive of delays in surgical healthcare but not for overall and non-surgical healthcare delays.

## 4. Discussion

To the best of our knowledge, this study is among the first to examine delayed surgical and non-surgical healthcare services as a result of the COVID-19 pandemic using a nationally representative sample of U.S. adults 50 years and older. Secondary analyses of HRS data indicated that >30% of U.S. adults ≥50 years delayed healthcare because of the COVID-19 pandemic. Moreover, having a history of 1 or 2 cardiovascular and/or metabolic conditions was associated with greater odds of delays in healthcare services, in general, and delays in non-surgical healthcare services, in particular, with distinct findings for histories of hypertension, cardiovascular disease, diabetes and stroke. It is worth noting that having 3 or more cardio-metabolic risk factors and/or chronic conditions was not associated with delayed healthcare services, an important finding suggesting that the highest-risk population in terms of multimorbidities had sought healthcare services or was able to receive needed services without delays. Being female, having a college degree or higher and 1-unit increase in depressive symptom score were key predictors of delays in surgical and/or non-surgical healthcare services. SuperLearner modeling was better able to predict surgical as opposed to overall and non-surgical delays in healthcare services. Although self-reported, delayed healthcare services may be the outcome of personal choice, system-level barriers, or both. 

Our results are consistent with those of similarly conducted published studies [65,66,81,82,83,84,85]. For instance, Chou et al. conducted a cohort study to evaluate the impact of COVID-19 on utilization of Emergency Department (ED) services among individuals (≥18 years) who were frequent ED users (≥4 ED visits per year) in Taiwan [82]. Researchers found that frequent ED users had shorter hospital stays in the ED during the COVID-19 pandemic (February 2020–January 2021) versus before the COVID-19 pandemic (February 2019–January 2020) and predictors of frequent ED use during the COVID-19 pandemic included triage level 4–5, pneumonia diagnosis, giddiness and dyspnea [82]. Nab et al. surveyed 1335 adult visitors of an ED at an academic hospital in the Netherlands using an online questionnaire and telephone interviews to examine the magnitude, characteristics and underlying motivations of delayed healthcare behavior during the first wave of the pandemic [84]. Their results suggest that nearly 20% of respondents delayed ED services, and of those, nearly 50% suggested that the COVID-19 pandemic had an influence on this delay [84]. These delays disproportionately affected older adults, those referred to the ED by the general practitioner or a medical specialist, and those visiting the ED with cardiac problems [84]. Frequently reported motives for delayed healthcare were “fear of contamination”, “not wanting to burden professionals”, “perceiving own complaints less urgent relative to COVID-19 patients”, “limited access to services”, and “stay home instructions from referring professionals” [84]. Using Johns Hopkins COVID-19 Civic Life and Public Health Survey data on 1337 U.S. adults, Anderson et al. estimated that 52% of individuals who needed care between March and mid-July 2020 had forgone care mainly owing to fear of SARS-CoV-2 transmission and financial concerns, with disparities according to race/ethnicity, socioeconomic status, age, and health status [65]. Using data on 155,825 non-elderly respondents (18–64 years) to three Urban Institute’s Household Pulse Survey waves (19–31 August, 14–26 October and 9–21 December 2020), Giannouchos et al., estimated the frequency of foregone and delayed care at 26.9% and 35.9%, respectively, with worse self-rated health status, increased mental health problems, Veterans Affairs or Medicaid coverage compared to private healthcare coverage, and older age identified as key predictors [66].

Although obesity is an established COVID-19 determinant [4,5,6], this study did not identify BMI as one of the key predictors of delaying surgical and/or non-surgical healthcare. By contrast, obesity-related multimorbidities was associated with delayed non-surgical but not surgical services, consistent with current evidence linking COVID-19 outcomes to cardiometabolic health [3,4,5,6,7,8,9,10]. Singh et al. published a systematic review and meta-analysis of 18 studies (14,558 COVID-19 patients), whereby estimated prevalence of comorbid conditions were 22.9% (95% CI: 15.8, 29.9) for hypertension, 11.5% (95% CI: 9.7, 13.4) for diabetes, 9.7% (95% CI: 6.8, 12.6) for cardiovascular disease and <4% for chronic obstructive pulmonary disease, chronic kidney disease, cerebrovascular disease and cancer [9]. COVID-19 severity was associated with most of these chronic conditions, except for cerebrovascular disease, while COVID-19 mortality increased for cardiovascular disease, chronic obstructive pulmonary disease, chronic kidney disease, cerebrovascular disease and cancer patients [9]. Finally, Liu et al., performed a systematic review and meta-analysis of 24 peer-reviewed articles (10,948 COVID-19 cases) whereby comorbid conditions were evaluated in relation to COVID-19 severity [86]. Prevalence rates of diabetes, coronary artery disease/cardiovascular disease, hypertension and chronic pulmonary disease were estimated at 10.0%, 8.0%, 20.0% and 3.0%, respectively; also, presence of comorbid conditions was strongly associated with COVID-19 severity (OR = 3.50, 95% CI: 1.78, 6.90) and being admitted to intensive care unit (OR = 3.36, 95% CI: 1.67, 6.76), but not with mortality (OR = 2.09, 95% CI: 0.26, 16.67) [86]. Accordingly, older adults with pre-existing cardiovascular and/or metabolic conditions may be more likely to take precautions, which include avoiding unnecessary COVID-19 exposure within healthcare settings. Bhatt et al. reported a decline in acute cardiovascular hospitalizations with shorter hospital stays among admitted patients, highlighting delayed, deferred, or abbreviated cardiovascular care during the initial phase of the pandemic [21].

Current evidence suggests that the COVID-19 pandemic has disproportionately affected specific groups including men [4,12,87], older adults [8,12,88,89], racial and ethnic minorities [3,4,7] as well as individuals of low socioeconomic status [13,90] and those having pre-existing chronic conditions [9,10,86]. The finding that women rather than men were more likely to delay healthcare as a result of the COVID-19 pandemic may be attributed to sex-specific social, psychological and behavioral factors that result in women being more likely than men to be compliant with COVID-19 restrictions and potentially less likely to contract COVID-19, as previously described by the Centers for Disease Control and Prevention [4,12,87]. Although aging may negatively impact host immunity thereby rendering those of advanced age at increased risk for infectious diseases, in general, and COVID-19, in particular, the two variables relevant to aging–age and HRS cohort–were not among the key predictors of delayed healthcare services as a result of the COVID-19 pandemic [4,10,88,89]. An unexpected finding was that there were no racial and ethnic disparities in terms of delayed healthcare, whereas several socioeconomic, lifestyle and health characteristics previously shown to differ among racial and ethnic groups and that may be indicators of health consciousness were predictive of delayed healthcare [3]. Higher education and its associated lifestyle and health characteristics may be markers of COVID-19 awareness, which can lead to behavioral change in the context of the COVID-19 pandemic, including delayed surgical and/or non-surgical healthcare. Finally, fair or poor self-rated health as well as depressive symptoms may constitute barriers among older adults for seeking and obtaining healthcare during the COVID-19 pandemic.

Interpretation of study findings need to take several limitations into account. First, linkage of the 2006–2018 and the 2020 COVID-19 HRS data as well as missing data on important variables may have resulted in analytic samples that were much smaller than the full sample of HRS with the potential for selection bias. Second, delays in healthcare and its hypothesized predictors were self-reported, which may lead to non-differential misclassification. Third, limited sample size may have impacted results pertaining to delayed surgical services and prevented in-depth examination of reasons for delayed healthcare. Additionally, nursing home and community residents were not distinguished within these analyses given the small sample size and the preliminary nature of the 2020 COVID-19 project data. Fourth, analyses were conducted using observational data and, as such, estimated relationships are prone to residual confounding and cannot be interpreted as being causal in nature. Fifth, the HRS sample consists of community and nursing home residents whose characteristics may differ systematically from COVID-19 patients identified in a clinical setting, precluding comparisons with the published literature [81,82,83,84,85,91]. In addition, 2020 COVID-19 project HRS data collection occurred during a specific time period in the absence of safe and efficacious vaccines and results obtained could not be extrapolated to later COVID-19 waves. Although predictors were assessed at multiple time points, the outcomes of interest were only assessed once. As such, we could not evaluate changes in delayed healthcare services as the pandemic continued, vaccines were introduced, and more information became publicized about COVID-19 risk and protective factors. Finally, secondary analysis of HRS data were performed and predictors covered by the 2006–2020 waves of HRS data may not be the most relevant as suggested by the SuperLearner model. Given the nature of the COVID-19 pandemic, future studies should simultaneously evaluate personal, family and community level predictors of healthcare access and utilization.

## 5. Conclusions

Among older adults, being female, having a college degree or higher and 1-unit increase in depression score were key predictors of delayed healthcare services attributed to the COVID-19 pandemic. Additionally, delays in surgical and non-surgical healthcare services may have distinct predictors with non-surgical delays more frequently observed among individuals with a history of 1 or 2 cardiovascular and/or metabolic conditions. COVID-19 messaging along with enhanced healthcare access through telehealth should target high-risk populations and especially those with chronic conditions. Additional studies are needed to elucidate the biopsychosocial mechanisms by which COVID-19 determinants might influence healthcare-related behaviors. Future studies should also focus on understanding the reasons for delayed healthcare as well as micro- and macro-level characteristics that could potentially affect an individual’s ability to obtain healthcare services, including level of concern with the pandemic, local transmission levels, stay at home orders and healthcare service closure or appointment cancellations due to surge capacity or staffing issues. These characteristics in addition to COVID-19 determinants can inform future public health interventions to prevent delayed surgical and/or non-surgical healthcare delivery among high-risk populations.

## Figures and Tables

**Table 1:** Baseline characteristics by healthcare service delays–HRS COVID-19 half-sample (N = 1413) ^a^.

	Total	Healthcare Service Delays		
		Overall (N = 1413)	Surgical (N = 1413)	Non-Surgical (N = 1413)
	% or Mean ± SEM	% OR (95% CI)	% OR (95% CI)	% OR (95% CI)
**OVERALL:**	100	32.7	5.8	31.4
**DEMOGRAPHIC/SOCIOECONOMIC:**				
***Sex***:		*p* = 0.017	*p* = 0.15	*p* = 0.023
Male	42.8	27.8 Ref.	7.3 Ref.	26.7 Ref.
Female	57.2	36.4 1.48 (1.07, 2.06)	4.7 0.63 (0.33, 1.18)	34.9 1.47 (1.05, 2.05)
***Birth cohort:***		*p* = 0.0002	*p* = 0.04	*p* = 0.0001
Original/AHEAD/Children of the Depression	14.9	22.7 Ref.	2.6 Ref.	20.9 Ref.
War Babies	13.9	25.4 1.16 (0.69, 1.94)	4.5 1.72 (0.54, 5.46)	24.9 1.25 (0.74, 2.10)
Early Baby Boomers	17.6	27.2 1.27 (0.79, 2.02)	3.8 1.43 (0.51, 4.06)	25.4 1.28 (0.79, 2.07)
Mid Baby Boomers	25.9	35.2 1.84 (1.21, 2.80)	8.7 3.52 (1.51, 8.22)	34.2 1.96 (1.27, 3.00)
Late Baby Boomers	27.5	43.0 2.56 (1.67, 3.93)	6.8 2.69 (1.07, 6.80)	41.5 2.67 (1.72, 4.12)
***Age (years):***		*p* < 0.0001	*p* = 0.02	*p* < 0.0001
Mean ± SEM	67.5 ± 0.3	0.96 (0.95, 0.98)	0.96 (0.93, 0.99)	0.96 (0.95, 0.98)
		*p* = 0.003	*p* = 0.01	*p* = 0.002
50–54	1.2	56.1 Ref.	0.0 Ref.	56.1 Ref.
55–59	20.0	42.1 0.57 (0.09, 3.48)	8.1 3.02 (1.11, 8.20)	40.8 0.54 (0.09, 3.31)
60–64	25.0	34.7 0.42 (0.07, 2.53)	7.1 2.61 (1.06, 6.45)	33.2 0.39 (0.06, 2.37)
65–69	17.2	34.4 0.41 (0.07, 2.51)	6.7 2.46 (0.92, 6.59)	33.1 0.39 (0.06, 2.37)
70–74	14.9	24.8 0.26 (0.04, 1.59)	5.7 2.07 (0.71, 6.02)	23.9 0.25 (0.04, 1.53)
75–79	8.3	26.4 0.28 (0.04, 1.78)	1.0 0.34 (0.08, 1.38)	25.6 0.27 (0.04, 1.72)
≥80	13.3	23.3 0.24 (0.04, 1.44)	2.8 --	21.4 0.21 (0.04, 1.29)
***Race:***		*p* = 0.61	*p* = 0.28	*p* = 0.63
White / Caucasian	76.5	33.2 Ref.	6.5 Ref.	32.1 Ref.
Black / African American	14.2	32.9 0.98 (0.69, 1.41)	4.0 0.60 (0.22, 1.62)	30.2 0.91 (0.63, 1.31)
Other	9.3	27.6 0.77 (0.45, 1.29)	3.2 0.47 (0.16, 1.40)	27.1 0.78 (0.46, 1.33)
***Ethnicity:***		*p* = 0.79	*p* = 0.04	*p* = 0.74
Hispanic	9.7	31.5 0.94 (0.61, 1.46)	2.5 0.38 (0.15, 0.97)	29.9 0.93 (0.39, 1.45)
Non-Hispanic	90.3	32.8 Ref.	6.2 Ref.	31.5 Ref.
***Education:***		*p* = 0.04	*p* = 0.24	*p* = 0.016
No degree	13.8	26.7 Ref.	4.3 Ref.	23.8 Ref.
GED	6.4	35.8 1.53 (0.72, 3.24)	14.4 3.76 (1.00, 14.11)	35.8 1.79 (0.84, 3.81)
High school diploma	27.5	30.2 1.19 (0.74, 1.92)	4.2 0.99 (0.37, 2.64)	29.3 1.33 (0.81, 2.18)
Some college	29.0	29.8 1.17 (0.72, 1.88)	5.3 1.25 (0.48, 3.22)	28.3 1.27 (0.77, 2.08)
College degree or higher	23.3	41.9 1.97 (1.22, 3.19)	7.0 1.69 (0.67, 4.26)	40.9 2.22 (1.36, 3.64)
***Marital status:***		*p* = 0.23	*p* = 0.63	*p* = 0.37
Never married	8.0	32.4 Ref.	4.1 Ref.	31.4 Ref.
Married/Partnered	56.3	31.3 0.95 (0.49, 1.83)	5.1 1.26 (0.31, 5.07)	30.1 0.94 (0.48, 1.82)
Separated/Divorced	20.2	39.5 1.36 (0.68, 2.75)	7.7 1.97 (0.44, 8.74)	37.2 1.29 (0.63, 2.63)
Widowed	15.4	29.0 0.85 (0.42, 1.75)	6.9 1.72 (0.34, 8.77)	28.5 0.87 (0.42, 1.80)
***Work status:***		*p* = 0.03	*p* = 0.66	*p* = 0.02
Working	33.5	38.3 1.46 (1.04, 2.05)	6.4 1.17 (0.58, 2.39)	37.3 1.49 (1.06, 2.11)
Not working	66.5	29.8 Ref.	5.5 Ref.	28.4 Ref.
***Federal health insurance coverage:***		*p* = 0.14	*p* = 0.34	*p* = 0.10
Yes	69.0	30.8 0.76 (0.54, 1.08)	5.2 0.71 (0.35, 1.44)	29.4 0.75 (0.52, 1.07)
No	30.9	36.9 Ref.	7.2 Ref.	35.8 Ref.
***Total wealth (USD):***		*p* = 0.79	*p* = 0.82	*p* = 0.60
<25,000	32.4	30.5 Ref.	6.1 Ref.	28.6 Ref.
25,000–124,999	51.7	33.4 1.14 (0.82, 1.59)	5.7 0.92 (0.46, 1.86)	32.8 1.22 (0.87, 1.71)
125,000–299,999	11.9	33.9 1.17 (0.69, 1.98)	4.3 0.69 (0.23, 2.10)	30.9 1.12 (0.65, 1.91)
≥300,000	3.9	37.5 1.37 (0.57, 3.27)	9.4 1.58 (0.33, 7.52)	37.5 1.49 (0.63, 3.58)
***Number of household members:***		*p* = 0.47	*p* = 0.95	*p* = 0.44
Mean ± SEM	2.2 ± 0.04	1.04 (0.93, 1.17)	1.01 (0.76, 1.33)	1.05 (0.93, 1.18)
		*p* = 0.39	*p* = 0.62	*p* = 0.30
≤3	88.3	32.2 Ref.	5.7 Ref.	30.8 Ref.
>3	11.6	36.6 1.22 (0.77, 1.92)	6.9 1.25 (0.51, 3.04)	36.1 1.27 (0.81, 1.99)
***Census region of residence:***		*p* = 0.25	*p* = 0.84	*p* = 0.18
Northeast	15.7	29.4 Ref.	4.8 Ref.	27.1 Ref.
Midwest	22.2	37.9 1.47 (0.86, 2.51)	7.1 1.52 (0.48, 4.79)	36.8 1.57 (0.91, 2.68)
South	41.1	29.7 1.01 (0.64, 1.60)	5.2 1.09 (0.40, 2.93)	28.4 1.07 (0.67, 1.72)
West	20.9	35.3 1.31 (0.79, 2.20)	6.4 1.34 (0.46, 3.93)	34.8 1.44 (0.85, 2.43)
**LIFESTYLE**:				
***Smoking status:***		*p* = 0.38	*p* = 0.06	*p* = 0.36
Never smoker	40.8	34.2 Ref.	4.4 Ref.	33.3 Ref.
Past smoker	43.8	30.0 0.82 (0.59, 1.14)	5.2 1.20 (0.59, 2.43)	28.6 0.80 (0.57, 1.12)
Current smoker	15.4	36.2 1.09 (0.67, 1.77)	11.3 2.76 (1.16, 6.56)	34.1 1.03 (0.62, 1.70)
***Frequency of alcohol consumption:***		*p* = 0.05	*p* = 0.09	*p* = 0.02
Abstinent	42.6	28.8 Ref.	4.5 Ref.	27.3 Ref.
1–3 days per month	18.2	42.5 1.82 (1.17, 2.80)	10.2 2.41 (1.06, 5.47)	41.9 1.92 (1.24, 2.96)
1–2 days per week	23.0	30.9 1.10 (0.74, 1.65)	3.7 0.82 (0.33, 2.01)	29.6 1.12 (0.74, 1.68)
≥3 days per week	16.1	34.3 1.29 (0.81, 2.05)	7.3 1.67 (0.70, 3.95)	32.8 1.30 (0.81, 2.08)
***Frequency of moderate/vigorous physical exercise:***		*p* = 0.77	*p* = 0.75	*p* = 0.86
Never	20.3	30.5 Ref.	4.6 Ref.	29.6 Ref.
1–4 times per month	25.1	34.2 1.18 (0.75, 1.87)	6.4 1.42 (0.55, 3.62)	31.5 1.09 (0.69, 1.75)
>1 times per week	54.6	32.8 1.11 (0.74, 1.67)	6.0 1.34 (0.56, 3.21)	32.0 1.12 (0.74, 1.69)
**HEALTH:**				
***Body mass index (kg/m^2^):***		*p* = 0.13	*p* = 0.72	*p* = 0.22
Mean ± SEM	30.4 ± 0.4	1.01 (0.99, 1.02)	1.00 (0.99, 1.01)	1.01 (0.99, 1.02)
		*p* = 0.87	*p* = 0.23	*p* = 0.92
<25	24.0	34.2 Ref.	4.1 Ref.	32.6 Ref.
25–29.9	32.8	31.9 0.90 (0.61, 1.34)	4.8 1.17 (0.47, 2.94)	31.1 0.93 (0.62, 1.40)
≥30	43.1	32.4 0.92 (0.63, 1.36)	7.6 1.89 (0.81, 4.43)	30.9 0.92 (0.62, 1.37)
***Cardiovascular and/or metabolic conditions:***				
*Hypertension:*		*p* = 0.77	*p* = 0.50	*p* = 0.82
Yes	62.9	33.1 1.05 (0.75, 1.46)	6.3 1.26 (0.64, 2.46)	31.7 1.04 (0.74, 1.46)
No	37.1	31.9 Ref.	5.1 Ref.	30.9 Ref.
*Diabetes:*		*p* = 0.30	*p* = 0.10	*p* = 0.27
Yes	28.1	35.5 1.19 (0.85, 1.67)	8.3 1.77 (0.89, 3.52)	34.4 1.21 (0.86, 1.70)
No	71.9	31.6 Ref.	4.9 Ref.	30.2 Ref.
*Heart disease:*		*p* = 0.74	*p* = 0.75	*p* = 0.84
Yes	29.6	31.8 0.95 (0.68, 1.31)	5.4 0.89 (0.46, 1.76)	30.8 0.97 (0.69, 1.34)
No	70.4	33.0 Ref.	6.0 Ref.	31.6 Ref.
*Stroke:*		*p* = 0.06	*p* = 0.68	*p* = 0.08
Yes	10.6	33.8 0.60 (0.35, 1.03)	4.8 0.79 (0.26, 2.42)	22.9 0.62 (0.36, 1.07)
No	89.4	23.5 Ref.	5.9 Ref.	32.4 Ref.
*Number of conditions:*		*p* = 0.21	*p* = 0.61	*p* = 0.28
0	25.0	29.3 Ref.	5.2 Ref.	28.0 Ref.
1–2	60.4	35.1 1.30 (0.87, 1.94)	5.6 1.07 (0.49, 2.32)	33.6 1.30 (0.86, 1.96)
≥3	14.1	28.3 0.95 (0.56, 1.60)	7.9 1.55 (0.59, 4.09)	27.9 0.99 (0.59, 1.69)
***Self-rated health:***		*p* = 0.03	*p* = 0.16	*p* = 0.07
Excellent/very good/good	64.1	29.8 Ref.	4.9 Ref.	28.9 Ref.
Fair/poor	35.8	37.8 1.43 (1.03, 1.97)	7.5 1.58 (0.83, 3.03)	35.7 1.36 (0.98, 1.89)
***Depression symptoms score:***		*p* = 0.0002	*p* = 0.01	*p* = 0.0005
Mean ± SEM	2.5 ± 0.07	1.15 (1.07, 1.24)	1.17 (1.04, 1.33)	1.14 (1.06, 1.24)

^a^ Odds ratios with their 95% confidence intervals were calculated using univariate logistic regression whereby delayed healthcare was modeled against each demographic, socioeconomic, lifestyle and health characteristic, with reported *p*-values corresponding to design-based F tests. Abbreviations: AHEAD = Study of Asset and Health Dynamics of the Oldest Old; COVID-19 = coronavirus disease 19; HRS = Health and Retirement Study; SEM = Standard error of the mean.

**Table 2:** Logistic regression models for cardiovascular and/or metabolic conditions as predictors of healthcare delays–HRS COVID-19 half-sample ^a^.

	Models I ^b^		Models II ^c^		Models III ^d^	
	OR	95% CI	OR	95% CI	OR	95% CI
*Logistic* (N = 1413):						
Body mass index (continuous)	1.00	0.99, 1.02	1.00	0.99, 1.02	1.01	0.99, 1.02
Body mass index (categorical):						
<25	Ref.		Ref.		Ref.	
25–29.9	0.86	0.57, 1.30	0.86	0.56, 1.31	0.95	0.62, 1.45
≥30	0.76	0.51, 1.14	0.74	0.49, 1.13	0.78	0.51, 1.19
Hypertension	1.34	0.93, 1.93	1.33	0.92, 1.91	1.27	0.87, 1.85
Diabetes	1.31	0.93, 1.83	1.26	0.89, 1.77	1.22	0.86, 1.74
Heart disease	1.17	0.82, 1.65	1.15	0.81, 1.65	1.02	0.69, 1.51
Stroke	0.74	0.43, 1.29	0.73	0.42, 1.28	0.57	0.32, 1.02
Number of cardiovascular and/or metabolic conditions:						
0	Ref.		Ref.		Ref.	
1–2	1.73	1.13, 2.65	1.74	1.15, 2.65	1.62	1.06, 2.49
≥3	1.44	0.82, 2.51	1.34	0.76, 2.36	1.15	0.63, 2.10
*Mixed effects logistic* (N = 2082):						
Body mass index (continuous)	1.00	1.00, 1.02	1.01	0.99, 1.02	1.00	0.99, 1.01
Body mass index (categorical):						
<25	Ref.		Ref.		Ref.	
25–29.9	1.09	0.94, 1.25	1.09	0.93, 1.27	1.08	0.93, 1.26
≥30	1.09	0.94, 1.26	1.07	0.92, 1.26	1.03	0.88, 1.20
Hypertension	0.92	0.82, 1.04	0.91	0.80, 1.03	0.85	0.75, 0.96
Diabetes	1.21	1.05, 1.39	1.24	1.08, 1.44	1.17	1.01, 1.36
Heart disease	1.14	0.99, 1.31	1.13	0.98, 1.30	1.01	0.87, 1.16
Stroke	0.90	0.72, 1.14	0.89	0.70, 1.14	0.81	0.63, 1.04
Number of cardiovascular and/or metabolic conditions:						
0	Ref.		Ref.		Ref.	
1–2	1.05	0.92, 1.19	1.07	0.93, 1.23	0.99	0.75, 1.31
≥3	1.19	0.96, 1.49	1.19	0.94, 1.49	0.99	0.77, 1.26

^a^ Odds ratios with their 95% confidence intervals were calculated using logistic or mixed effects logistic regression models for each cardiovascular and/or metabolic condition as a predictor of surgical and/or non-surgical healthcare delay. ^b^ Models I are adjusted for demographic and socioeconomic characteristics; ^c^ Models II are adjusted for demographic, socioeconomic and lifestyle characteristics; ^d^ Models III are adjusted for demographic, socioeconomic, lifestyle and health characteristics. Abbreviations: CI = Confidence interval; COVID-19 = Coronavirus disease 19; HRS = Health and Retirement Study; OR = Odds ratio.

**Table 3:** Logistic regression models for cardiovascular and/or metabolic conditions as predictors of surgical delays–HRS COVID-19 half-sample ^a^.

	Models I ^b^		Models II ^c^		Models III ^d^	
	OR	95% CI	OR	95% CI	OR	95% CI
*Logistic* (N = 1413):						
Body mass index (continuous)	0.99	0.98, 1.01	1.00	0.99, 1.01	1.00	0.98, 1.01
Body mass index (categorical):						
<25	Ref.		Ref.		Ref.	
25–29.9	1.05	0.43, 2.55	1.19	0.44, 3.22	1.22	0.45, 3.28
≥30	1.64	0.72, 3.72	1.93	0.77, 4.88	1.83	0.72, 4.63
Hypertension	1.40	0.69, 2.86	1.33	0.65, 2.73	1.18	0.55, 2.52
Diabetes	1.62	0.86, 3.04	1.52	0.80, 2.88	1.43	0.76, 2.68
Heart disease	0.84	0.44, 1.60	0.79	0.42, 1.48	0.67	0.35, 1.29
Stroke	0.79	0.27, 2.27	0.72	0.26, 1.98	0.62	0.21, 1.83
Number of cardiovascular and/or metabolic conditions:						
0	Ref.		Ref.		Ref.	
1–2	1.22	0.54, 2.74	1.24	0.53, 2.89	1.14	0.48, 2.69
≥3	1.64	0.59, 4.52	1.34	0.49, 3.68	1.10	0.39, 3.15
*Mixed effects logistic* (N = 2082):						
Body mass index (continuous)	1.02	1.00, 1.04	1.01	0.99, 1.04	1.01	0.98, 1.02
Body mass index (categorical):						
<25	Ref.		Ref.		Ref.	
25–29.9	1.08	0.75, 1.55	1.17	0.79, 1.72	1.17	0.79, 1.71
≥30	1.13	0.79, 1.60	1.03	0.70, 1.52	0.93	0.63, 1.38
Hypertension	0.84	0.63, 1.09	0.79	0.59, 1.04	0.69	0.52, 0.92
Diabetes	1.73	1.28, 2.34	1.59	1.18, 2.16	1.42	1.04, 1.92
Heart disease	1.02	0.75, 1.39	0.88	0.64, 1.20	0.69	0.49, 0.98
Stroke	2.40	0.98, 5.91	1.95	1.29, 2.91	1.69	1.11, 2.57
Number of cardiovascular and/or metabolic conditions:						
0	Ref.		Ref.		Ref.	
1–2	0.98	0.69, 1.38	0.91	0.64, 1.28	0.80	0.56, 1.15
≥3	2.49	1.62, 1.38	1.87	1.21, 2.92	1.36	0.85, 2.19

^a^ Odds ratios with their 95% confidence intervals were calculated using logistic or mixed effects logistic regression models for each cardiovascular and/or metabolic condition as a predictor of surgical healthcare delay. ^b^ Models I are adjusted for demographic and socioeconomic characteristics; ^c^ Models II are adjusted for demographic, socioeconomic and lifestyle characteristics; ^d^ Models III are adjusted for demographic, socioeconomic, lifestyle and health characteristics. Abbreviations: CI = Confidence interval; COVID-19 = Coronavirus disease 19; HRS = Health and Retirement Study; OR = Odds ratio.

**Table 4:** Logistic regression models for cardiovascular and/or metabolic condition as predictors of non-surgical delays–HRS COVID-19 half-sample ^a^.

	Models I ^b^		Models II ^c^		Models III ^d^	
	OR	95% CI	OR	95% CI	OR	95% CI
*Logistic* (N = 1413):						
Body mass index (continuous)	1.00	0.99, 1.01	1.01	0.99, 1.02	1.00	0.99, 1.01
Body mass index (categorical):						
<25	Ref.		Ref.		Ref.	
25–29.9	0.89	0.58, 1.36	0.89	0.58, 1.36	0.97	0.63, 1.48
≥30	0.76	0.50, 1.14	0.74	0.48, 1.13	0.76	0.50, 1.18
Hypertension	1.35	0.93, 1.94	1.34	0.93, 1.94	1.30	0.89, 1.89
Diabetes	1.32	0.94, 1.87	1.26	0.89, 1.79	1.22	0.85, 1.75
Heart disease	1.21	0.85, 1.73	1.21	0.84, 1.74	1.09	0.74, 1.62
Stroke	0.78	0.45, 1.35	0.77	0.44, 1.34	0.59	0.33, 1.06
Number of cardiovascular and/or metabolic conditions:						
0	Ref.		Ref.		Ref.	
1–2	1.75	1.14, 2.69	1.79	1.16, 2.72	1.68	1.08, 2.59
≥3	1.55	0.88, 2.73	1.46	0.83, 2.58	1.26	0.69, 2.32
*Mixed effects logistic* (N = 2082):						
Body mass index (continuous)	1.01	0.99, 1.02	1.00	0.99, 1.02	1.00	0.99, 1.01
Body mass index (categorical):						
<25	Ref.		Ref.		Ref.	
25–29.9	1.09	0.94, 1.26	1.08	0.93, 1.27	1.09	0.93, 1.27
≥30	1.06	0.91, 1.22	1.05	0.89, 1.23	1.01	0.86, 1.19
Hypertension	0.87	0.77, 0.98	0.87	0.77, 0.99	0.81	0.71, 0.92
Diabetes	1.17	1.01, 1.35	1.20	1.04, 1.39	1.13	0.97, 1.31
Heart disease	1.15	1.00, 1.32	1.14	0.99, 1.32	1.03	0.89, 1.19
Stroke	0.88	0.69, 1.11	0.88	0.68, 1.13	0.79	0.62, 1.02
Number of cardiovascular and/or metabolic conditions:						
0	Ref.		Ref.		Ref.	
1–2	1.00	0.88, 1.15	1.04	0.90, 1.19	0.96	0.84, 1.11
≥3	1.09	0.87, 1.37	1.11	0.87, 1.40	0.92	0.72, 1.18

^a^ Odds ratios with their 95% confidence intervals were calculated using logistic or mixed effects logistic regression models for each cardiovascular and/or metabolic condition as a predictor of non-surgical healthcare delay. ^b^ Models I are adjusted for demographic and socioeconomic characteristics; ^c^ Models II are adjusted for demographic, socioeconomic and lifestyle characteristics; ^d^ Models III are adjusted for demographic, socioeconomic, lifestyle and health characteristics. Abbreviations: CI = Confidence interval; COVID-19 = Coronavirus disease 19; HRS = Health and Retirement Study; OR = Odds ratio.

**Table 5:** Logistic regression models for key predictors of delayed healthcare–HRS COVID-19 half-sample ^a^.

*Predictors:*	Logistic Regression Models			Mixed-Effects Logistic Regression Models		
	Overall	Surgical	Non-Surgical	Overall	Surgical	Non-Surgical
	OR (95% CI)	OR (95% CI)	OR (95% CI)	OR (95% CI)	OR (95% CI)	OR (95% CI)
***Sex:***						
Male	Ref.	Ref.	Ref.	Ref.	Ref.	Ref.
Female	1.44 (1.02, 2.02)	0.59 (0.32, 1.14)	1.42 (1.01, 2.00)	1.34 (1.18, 1.53)	0.51 (0.38, 0.67)	1.35 (1.19, 1.54)
***Age (years):***	0.97 (0.95, 0.99)	0.97 (0.94, 1.00)	0.97 (0.95, 0.99)	0.98 (0.97, 0.99)	0.96 (0.94, 0.97)	0.98 (0.97, 0.99)
***Ethnicity:***						
Hispanic	--	0.35 (0.13, 0.92)	--	--	0.29 (0.18, 0.49)	--
Non-Hispanic	Ref.	Ref.	Ref.	Ref.	Ref.	Ref.
***Education:***						
No degree	Ref.	Ref.	Ref.	Ref.	Ref.	Ref.
GED	1.25 (0.61, 2.55)	--	1.46 (0.71, 3.00)	1.33 (0.98, 1.79)	--	1.43 (1.06, 1.94)
High school diploma	1.11 (0.68, 1.83)	--	1.23 (0.74, 2.07)	1.03 (0.84, 1.26)	--	1.04 (0.85, 1.28)
Some college	1.03 (0.63, 1.70)	--	1.14 (0.68, 1.90)	1.24 (1.01, 1.52)	--	1.28 (1.04, 1.57)
College degree or higher	1.94 (1.14, 3.27)	--	2.14 (1.26, 3.66)	1.77 (1.44, 2.17)	--	1.80 (1.46, 2.22)
***Work status:***						
Working	1.38 (0.89, 2.11)	--	1.35 (0.87, 2.08)	1.21 (1.04, 1.39)	--	1.19 (1.03, 1.38)
Not working	Ref.	Ref.	Ref.	Ref.	Ref.	Ref.
***Federal health insurance:***						
Yes	1.14 (0.71, 1.82)	--	1.10 (0.68, 1.78)	1.08 (0.90, 1.28)	--	1.11 (0.94, 1.32)
No	Ref.	Ref.	Ref.	Ref.	Ref.	Ref.
***Smoking status:***						
Never smoker	Ref.	Ref.	Ref.	Ref.	Ref.	Ref.
Past smoker	--	1.04 (0.50, 2.15)	--	--	2.04 (1.50, 2.77)	--
Current smoker	--	1.88 (0.82, 4.33)	--	--	1.97 (1.28, 3.03)	--
***Alcohol consumption:***						
Abstinent	Ref.	Ref.	Ref.	Ref.	Ref.	Ref.
1–3 days per month	1.79 (1.15, 2.77)	2.39 (1.07, 5.32)	1.83 (1.18, 2.85)	1.10 (0.94, 1.29)	1.34 (0.91, 1.97)	1.09 (0.93, 1.29)
1–2 days per week	0.99 (0.64, 1.55)	0.74 (0.29, 1.86)	0.98 (0.63, 1.54)	1.14 (0.98, 1.34)	0.92 (0.63, 1.36)	1.13 (0.97, 1.32)
≥3 days per week	1.32 (0.81, 2.17)	1.78 (0.70, 4.51)	1.28 (0.78, 2.11)	1.19 (0.99, 1.43)	0.86 (0.54, 1.36)	1.19 (0.99, 1.44)
***Body mass index:***	1.00 (0.99, 1.01)	--	--	1.00 (0.99, 1.01)	--	--
***Self-rated health:***						
Excellent/very good/good	Ref.	Ref.	Ref.	Ref.	Ref.	Ref.
Fair/poor	1.47 (1.03, 2.11)	1.06 (0.56, 2.00)	1.41 (0.97, 2.04)	1.53 (1.31, 1.78)	1.60 (1.12, 2.27)	1.49 (1.27, 1.74)
***Depressive symptoms:***	1.14 (1.05, 1.23)	1.15 (0.99, 1.34)	1.14 (1.04, 1.23)	1.08 (1.04, 1.11)	1.12 (1.05, 1.20)	1.08 (1.04, 1.11)
***Diabetes:***						
Yes	--	1.57 (0.84, 2.90)	--	--	1.55 (1.09, 2.19)	--
No	Ref.	Ref.	Ref.	Ref.	Ref.	Ref.
***Stroke:***						
Yes	0.57 (0.32, 1.01)	--	0.59 (0.34, 1.05)	0.83 (0.65, 1.06)	--	0.80 (0.63, 1.03)
No	Ref.	Ref.	Ref.	Ref.	Ref.	Ref.

^a^ Odds ratios with their 95% confidence intervals were calculated using logistic or mixed effects logistic regression models for key predictors of healthcare delay. Abbreviations: CI = Confidence interval; COVID-19 = Coronavirus disease 19; HRS = Health and Retirement Study; OR = Odds ratio.

**Table 6:** SuperLearner models for predictors of healthcare delays–HRS COVID-19 half-sample (N = 1413).

	Healthcare Delays		
	Overall	Surgical	Non-Surgical
*Model A: LASSO*			
cv-Risk	0.214	0.052	0.210
*Model B: Random Forest*			
cv-Risk	0.226	0.054	0.218
*Model C: XGBOOST*			
cv-Risk	0.278	0.057	0.279
*Model D: SVM*			
cv-Risk	0.224	0.224	0.216
*Super Learner:*			
cv-Risk	0.214	0.052	0.211
AUC	0.600	0.920	0.655

Abbreviations: AUC = Area under the curve; COVID-19 = Coronavirus disease 19; cv = Cross-validated; HRS = Health and Retirement Study; LASSO = Least Absolute Shrinkage and Selection Operator; SVM = Support Vector Machines.

## Data Availability

The data that support the findings of this study are available from the Health and Retirement Study but restrictions apply to the availability of these data, which were used under license for the current study, and so are not publicly available. Data are however available from the authors upon reasonable request and with permission of the Health and Retirement Study.
